# Caught in a vicious cycle: Cell‐free hemoglobin and the pathogenesis of pulmonary hypertension

**DOI:** 10.1002/pul2.12194

**Published:** 2023-01-24

**Authors:** Janae Gonzales, Dustin R. Fraidenburg

**Affiliations:** ^1^ Department of Medicine University of Illinois at Chicago Chicago Illinois USA

Hemolysis and the toxicity of cell‐free hemoglobin (CFH) are well‐studied across several organ systems and lead to detrimental acute and chronic effects. The influence of hemolysis on the development of pulmonary hypertension (PH) has mostly been studied within the context of hematologic disorders, and more specifically in individuals with sickle cell disease (SCD). This complication affects nearly 10% of the SCD population and, of these patients, nearly half will have vascular remodeling and a phenotype similar to pulmonary arterial hypertension (PAH).^1^ In the past decade, investigators have uncovered a role for hemolysis in the pathophysiology of PAH in relation to the adverse effects of CFH, with exact mechanisms remaining incompletely understood. It is in this context that we read with great enthusiasm the article by Dr. Meegan and her colleagues on the role of transpulmonary generation of CFH as a novel mechanism for vascular dysfunction in PAH.^2^

In the current issue of *Pulmonary Circulation*, Meegan et al. identify a novel mechanism whereby CFH contributes to PAH pathology. A prospective cohort of PAH patients underwent right heart catheterization and plasma CFH levels were measured at the pulmonary artery and wedge positions. They demonstrated that CFH increases across the pulmonary microcirculation in individuals with PAH, suggesting that an in‐situ generation of CFH may contribute to or be a consequence of pulmonary vascular remodeling. These findings were positively correlated with severity of disease by measurements of mean pulmonary artery pressure and pulmonary vascular resistance. In a different subset of PAH patients, they also demonstrated increased hemoglobin α expression in pulmonary arterioles of explanted lungs when compared to healthy controls, and specifically increased expression in occluded vessels when compared to patent vessels. While the mean change in CFH across the pulmonary circulation is small in comparison to circulating levels at steady state, the location of intravascular hemolysis is particularly intriguing.

Lastly, the authors analyzed a large cohort to further explain the elevated CFH levels with particular focus on clearance mechanisms. They did not find any specific haptoglobin genotypic differences between PAH and control groups. The notable findings were lower circulating levels of haptoglobin and hemopexin in the hereditary PAH (HPAH) cohort, which could simply be explained by the increased binding to the elevated circulating CFH, but this significant change remained after correcting for CFH levels (CFH to haptoglobin ratio). Interestingly, they also evaluated unaffected BMPR2 mutation carriers and these individuals had significantly higher haptoglobin and hemopexin levels despite their prior study that demonstrated elevated circulating CFH in this group.^3^ Although no interventions were evaluated in this study, these observational findings identify the effective clearance of CFH in PAH as an important process that warrants further study.

During hemolysis, the rapid turnover of red blood cells releases hemoglobin into the plasma, which typically is scavenged by haptoglobin for clearance. An excess of CFH leads to tissue and vascular injury. CFH has notably been elevated in patients with SCD,^4^ preclinical PAH models,^5^ and individuals with PAH; and these concentrations correlate with disease severity, BMPR2 mutation, and adverse outcomes.^3^, ^5^ The incidence of PH in hemolytic disorders has largely been attributed to CFH and its propensity to scavenge nitric oxide (NO), depleting its bioavailability and causing vasoconstriction as well as platelet activation and generation of reactive oxygen species. Prior studies have demonstrated the development of PH in SCD and hemolytic anemia animal models, yet dysregulated NO is not associated with development of significant vascular remodeling.^6^ Targeting NO regulated vasodilation has been an important cornerstone of our current PH therapies, which focuses considerably on realigning the balance of vasodilatory and vasoconstrictive mechanisms in the pulmonary vasculature; however, a need remains for targeted therapies earlier in the disease process that reduce and prevent vascular injury and remodeling.

CFH leads to endothelial vascular injury through several mechanisms. CFH undergoes oxidation in the plasma which releases heme and generates reactive oxygen species leading to endothelial activation and inflammation. The effect of free heme and its oxidized moiety hemin has been studied in preclinical models of SCD and show the development of severe lung injury through toll‐like receptor 4 (TLR4) signaling and generation of neutrophil extracellular traps.^7^, ^8^ In vitro studies on the pulmonary vascular endothelium have demonstrated that free heme exposure leads to hyperpermeability, disruption of tight junctions, and necroptotic cell death.^5^, ^9^ A recent in vitro study by our group implicates free heme in the development of pulmonary artery endothelial hyperproliferation and dysfunction and identifies endothelial to mesenchymal transition (EndoMT) as a key mechanism.^10^ EndoMT is a process of cellular transdifferentiation where endothelial cells lose their barrier function and gain contractile mesenchymal properties. EndoMT is an established mechanism in the vascular remodeling seen in PAH and, as we have recently shown; free heme may be an important contributor to this process.

Taken together, these studies emphasize the importance of hemolysis and CFH in the development of PAH and PH due to chronic hemolysis. The influence of CFH on the pulmonary endothelium is a spectrum from endothelial dysfunction and vascular injury to barrier hyperpermeability and fatal lung injury. Individuals with chronic hemolytic states such as SCD experience a full range of these effects as seen in the prominent pulmonary complications of PH and acute chest syndrome. Meegan et al. findings of in‐situ CFH across the pulmonary circulation suggest that a pulmonary hemolytic process may be occurring in the absence of systemic hemolysis or as an additional injurious response. This suggests that hemolysis may play a role in both triggering endothelial dysfunction and causing ongoing progression of arterial remodeling after an inciting event. The authors note that the accumulation of hemoglobin immunostaining at occluded vessels supports the notion that free hemoglobin is released in areas of greatest vascular remodeling and potentially creating a feed‐forward mechanism. In conjunction with our groups recent study, the continued release of free heme on the pulmonary microcirculation would likely contribute to further vascular remodeling in the pulmonary arterioles via EndoMT and endothelial dysfunction. Additionally, the potential lack of clearance of free heme further prolongs exposure of the pulmonary endothelium to free heme and allows for a vicious cycle of injury, vasoconstriction, inflammation, and more hemolysis (Figure 1). This new study broadens the importance of CFH to all forms of PH and supports the need for further research targeting this modifiable insult.

**Figure 1:**
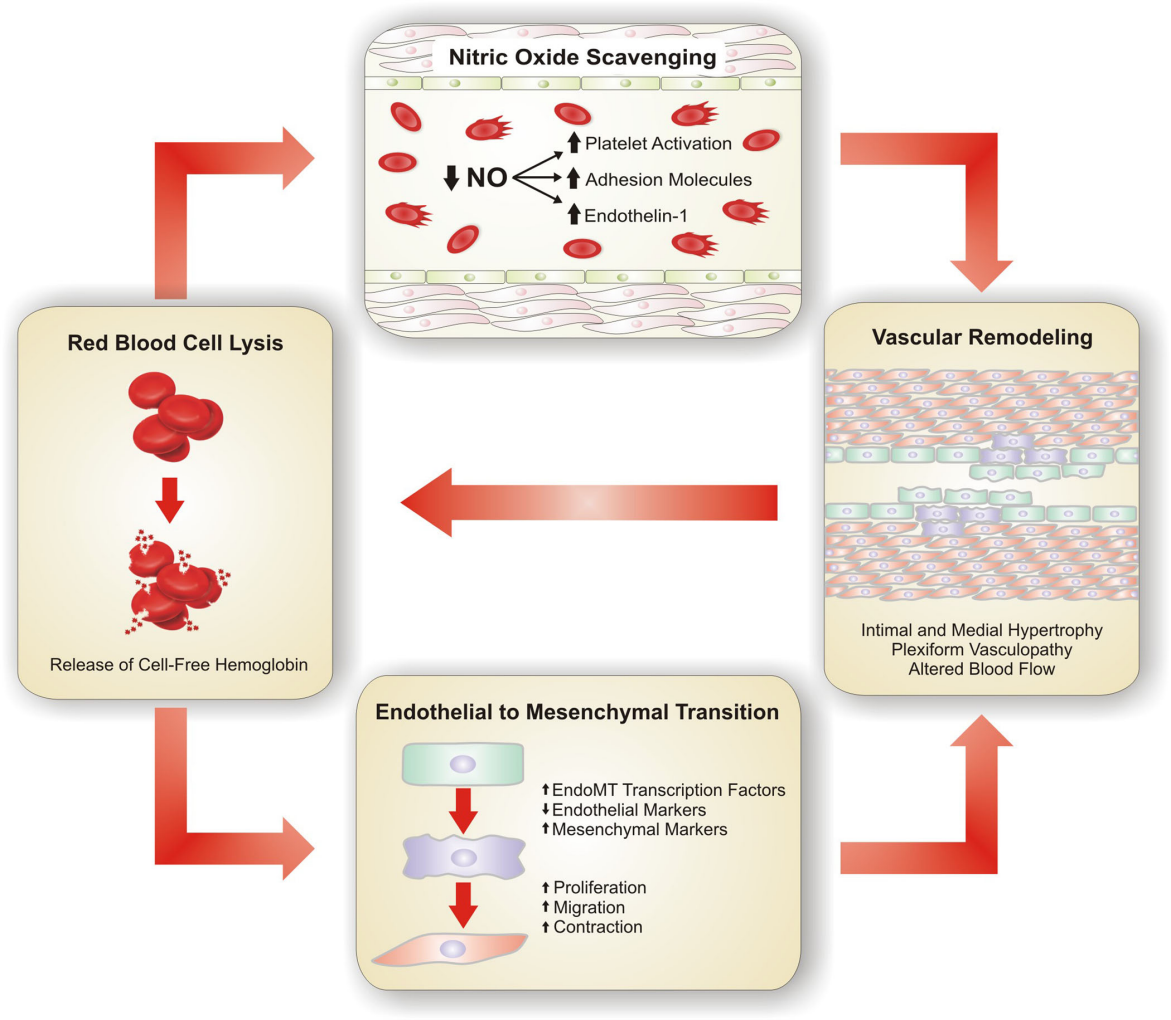
The vicious cycle of transpulmonary release of cell‐free hemoglobin. Red blood cell lysis and release of cell free hemoglobin leads to nitric oxide scavenging and endothelial dysfunction with endothelial to mesenchymal transition. These processes contribute to vascular remodeling with intimal and medial hypertrophy as well as plexiform vasculopathy causing turbulent blood flow and obstruction leading to further red blood cell lysis. NO, nitric oxide.

## AUTHOR CONTRIBUTIONS

Janae Gonzales and Dustin R. Fraidenburg both conceived and contributed to writing the manuscript.

## CONFLICT OF INTEREST STATEMENT

The authors declare no conflict of interest.

## ETHICS STATEMENT

This work does not involve human subjects, animal experimentation, or cell lines.
